# Novel ASK1 inhibitor AGI‐1067 improves AGE‐induced cardiac dysfunction by inhibiting MKKs/p38 MAPK and NF‐κB apoptotic signaling

**DOI:** 10.1002/2211-5463.12499

**Published:** 2018-08-17

**Authors:** Zhongwei Liu, Shixiang Zheng, Xi Wang, Chuan Qiu, Yan Guo

**Affiliations:** ^1^ Key Laboratory of Biomedical Information Engineering of Ministry of Education School of Life Science and Technology Xi'an Jiaotong University China; ^2^ Department of Cardiology Shaanxi Provincial People's Hospital Xi'an China; ^3^ Department of Vascular Surgery Brigham and Women's Hospital Boston MA USA; ^4^ Department of Critical Care Medicine Union Hospital of Fujian Medical University Fuzhou China; ^5^ Department of Obstetrics and Gynecology The Second Xiangya Hospital Central South University Changsha China; ^6^ Department of Biostatistics & Bioinformatics School of Public Health & Tropical Medicine Tulane University New Orleans LA USA

**Keywords:** advanced glycation end products, apoptosis, apoptosis signal‐regulating kinase, diabetes, heart failure

## Abstract

Heart failure has been identified as one of the clinical manifestations of diabetic cardiovascular complications. Excessive myocardium apoptosis characterizes cardiac dysfunctions, which are correlated with an increased level of advanced glycation end products (AGEs). In this study, we investigated the participation of reactive oxygen species (ROS) and the involvements of apoptosis signal‐regulating kinase 1 (ASK1)/mitogen‐activated protein kinase (MAPK) kinases (MKKs)/p38 MAPK and nuclear factor κB (NF‐κB) pathways in AGE‐induced apoptosis‐mediated cardiac dysfunctions. The antioxidant and therapeutic effects of a novel ASK1 inhibitor, AGI‐1067, were also studied. Myocardium and isolated primary myocytes were exposed to AGEs and treated with AGI‐1067. Invasive hemodynamic and echocardiographic assessments were used to evaluate the cardiac functions. ROS formation was evaluated by dihydroethidium fluorescence staining. A terminal deoxynucleotidyl transferase dUTP nick end labelling assay was used to detect the apoptotic cells. ASK1 and NADPH activities were determined by kinase assays. The association between ASK1 and thioredoxin 1 (Trx1) was assessed by immunoprecipitation. Western blotting was used to evaluate the phosphorylation and expression levels of proteins. Our results showed that AGE exposure significantly activated ASK1/MKKs/p38 MAPK, which led to increased cardiac apoptosis and cardiac impairments. AGI‐1067 administration inhibited the activation of MKKs/p38 MAPK by inhibiting the disassociation of ASK1 and Trx1, which suppressed the AGE‐induced myocyte apoptosis. Moreover, the NF‐κB activation as well as the ROS generation was inhibited. As a result, cardiac functions were improved. Our findings suggested that AGI‐1067 recovered AGE‐induced cardiac dysfunction by blocking both ASK1/MKKs/p38 and NF‐κB apoptotic signaling pathways.

**Abbreviations**
AGE: advanced glycation end product
ASK: apoptosis signal‐regulating kinase
DAPI: 4′,6‐diamidino‐2‐phenylindole
DbCM: diabetic cardiomyopathy
DHE: dihydroethidium
GAPDH: glyceraldehyde 3‐phosphate dehydrogenase
GST: glutathione *S*‐transferase
IP: immunoprecipitation
LVEDD: left ventricular end‐diastole diameter
LVEDP: left ventricular end‐diastolic pressure
LVEF: left ventricular ejection fraction
LVESD: left ventricular end‐systole diameter
LVFS: left ventricular fractional shortening
LVSP: left ventricular systolic pressure
MAPK: mitogen‐activated protein kinase
MKK: MAPK kinase kinase
NF‐κB: nuclear factor κB
ROS: reactive oxygen species
Trx: thioredoxin
TUNEL: terminal deoxynucleotidyl transferase dUTP nick end labeling

The occurrence of diabetes mellitus has been growing fast in the past few decades globally. The morbidity and mortality make diabetes mellitus one of the prevailing public health issues. Acting as an independent risk factor, hyperglycemia plays a vital role in diabetic cardiovascular complications by inducing metabolic disorders 1. Myocardial infarction, ischemic cardiomyopathy, sudden cardiac death and diabetic cardiomyopathy (DbCM) are the most frequent clinical manifestations of diabetic cardiovascular complications 2. DbCM is a unique cardiomyopathy in diabetic patients characterized by impaired cardiac diastolic and systolic functions 3. Though the mechanisms concerned in DbCM are still unclear, our and others' previous investigations suggested that apoptosis‐caused myocyte contractile unit loss is responsible for the cardiac dysfunctions in DbCM.

During diabetes, sustained high blood glucose concentration leads to abnormally altered glucose metabolism. The glucose residues and their metabolites react with amino groups of proteins and certain nucleic acids through non‐enzymatic glycosylation reactions by which a group of high molecular mass fluorescent substances are generated. These substances were named advanced glycation end products (AGEs) 4. The AGE production increases dramatically in diabetes patients and is considered as an important pathogenic factor participating in the occurrence and development of cardiovascular disorders 5. It was reported that elevated blood AGE concentration was highly correlated with both diabetes and heart failure 6. Moreover, it was reported that AGEs induced apoptosis in the human myocyte H9c2 cell line 7. Thus, exploring novel agents targeting AGE‐induced apoptosis would be of therapeutic value.

As a member of the mitogen‐activated protein kinases (MAPKs) family, p38 MAPK is a key factor conducting apoptotic signaling 8. The activation of p38 MAPK is regulated by its upper stream kinases, namely MAPK kinase kinase (MKK) 3 and MKK6 9. As a MKK kinase, apoptosis signal‐regulating kinase (ASK) 1 mediates the activation of MKKs 10. A few previous studies drew to our attention that AGEs induce the activation of ASK1 by disassociating ASK1 from its intrinsic inhibitor, thioredoxin (Trx) 11. Therefore, we raised the hypothesis that as an apoptosis inducer, AGEs could cause death of myocytes by activating ASK1/MKKs/p38 MAPK signaling.

AGI‐1067, also called succinobucol, is a chemically synthesized derivative of probucol that showed antioxidant and anti‐inflammatory activities and the ability to regress coronary atherosclerosis 12. In this study, the attenuating effects of AGI‐1067 on AGE‐induced myocyte apoptosis and cardiac dysfunction, as well as the mechanisms, were investigated by using AGE‐treated animals. We believe that results from this study will not only add to our knowledge of the pathogenesis of DbCM, but also provide a theoretical basis for potential application of AGI‐1067 for DbCM treatment.

## Materials and methods

### AGEs–BSA preparation

AGEs–BSA was prepared according to the method described in our and others' previous investigations 9, 13. Briefly, glyceraldehyde (0.1 mmol·L^−1^; Sigma‐Aldrich, Burlington, MA, USA) was incubated with BSA (HyClone, Los Angeles, CA, USA) in sodium phosphate buffer (0.2 mmol·L^−1^, pH 7.4) under sterile conditions at 37 °C for 7 days. The unincorporated sugars were eliminated by chromatography with PD‐10 desalting columns (GE Healthcare, Stockholm, Sweden) and dialysis against PBS. The non‐glycated BSA was also prepared without glyceraldehyde and used as control.

### Animals and treatments

Sprague–Dawley (SD) rats (9 weeks old, weight 180–220 g) were purchased from the Experimental Animal Center of Xi'an Jiaotong University. Animals were raised in independent polypropylene cages in an environment providing appropriate temperature (25 ± 1 °C), humidity (65 ± 5%) and an artificial 12‐h light/dark cycle. The animals were free to consume sterilized water and standard chow. Experimental protocols were carried out by following the *Recommended Guidelines for the Care and Use of Laboratory Animals* issued by the Chinese Council on Animal Research. The animal experimental protocols were reviewed and approved by the Animal Ethics Committee of Xi'an Jiaotong University. Rats were exposed to AGEs by intraperitoneal injection of 1 mg of AGEs–BSA daily for 20 consecutive days. AGI‐1067 was administrated to rats by intraperitoneal injection at a dosage of 50, 100 and 150 mg·kg^−1^ bodyweight. Carbon dioxide asphyxia was applied to sacrifice the rats and the samples were harvested.

### Myocytes culture and treatment

Isolated primary myocytes were investigated in this study. The myocytes were isolated from neonate SD rats (2 days old, Animal Experimental Center of Xi'an Jiaotong University) according to our previous protocols. Briefly, hearts were harvested from the neonatal rats and further treated by Liberase perfusion (4.5 mg·mL^−1^; Roche, Amstel dam, Holland). Cells were cultured in minimum essential medium supplemented with Hanks' buffered salt solution (HyClone), bovine calf serum (5%; HyClone), l‐glutamine (2 mmol·L^−1^; Invitrogen, Carlsbad, CA, USA), CaCl_2_ (1.8 mmol·L^−1^), penicillin (100 U·mL^−1^; Sigma‐Aldrich), streptomycin (100 mg·mL^−1^; Sigma‐Aldrich), and 2,3‐butanedione monoxime (10 mmol·L^−1^; Sigma‐Aldrich) for 1 h. Then the medium was replaced with minimum essential medium containing myocyte BSA (0.1 mg·mL^−1^; Invitrogen), l‐glutamine (2 mmol·L^−1^; Invitrogen), penicillin (100 U·mL^−1^; Sigma‐Aldrich) and streptomycin (100 mg·mL^−1^; Sigma‐Aldrich) in a humidified incubator providing 5% CO_2_ and 95% fresh air at 37 °C. Cells at confluence of 90% were used for subsequent experiments. Cells were exposed to AGEs at a final concentration of 10 μmol·L^−1^ for 24 h. Several cells were co‐administrated with AGI‐1067 at concentrations of 5, 10 and 15 μmol·L^−1^.

### Cardiac function determination

Catheter‐based invasive hemodynamic assessment and echocardiography were used to evaluate the cardiac function in the animals in this study. The protocol was carried out in accordance with our previous studies 14. Rats were anesthetized by isoflurane inhalation. A Mikro‐Tip catheter (Millar, Chicago, IL, USA) was intubated into left ventricle through the right carotid artery. The pressure signal was sensed by a transducer and further analyzed with PowerLab 4/25 Biological Analysis System (ADInstruments, Chicago, IL, USA). The left ventricular systolic pressure (LVSP), left ventricular end‐diastolic pressure (LVEDP), maximal rate of left ventricular increased pressure (+LVd*P*/d*t*) and maximal rate of left ventricular decreased pressure (−LVd*P*/d*t*) were recorded and measured. The hair of the anterior chest was removed. Animals were placed left laterally. The echocardiographic examinations were carried out in accordance with our previous descriptions 15. The Vivid 7 dimension system (GE Healthcare) with a probe (12L) at 10 MHz was used. The probe was placed parallel to the left margin of the sternum after the image depth was adjusted ranging from 2.0 to 4.0 cm. Regurgitant jets were assessed by two‐dimensional color and continuous Doppler. M‐mode tracings were applied to determine the diameters of cardiac chambers. Left ventricular end‐systole diameter (LVESD), left ventricular end‐diastole diameter (LVEDD), left ventricular ejection fraction (LVEF) and left ventricular fractional shortening (LVFS) were determined and recorded.

### *In situ* apoptosis determination

The apoptosis of myocardium and cultured myocytes was detected by terminal deoxynucleotidyl transferase dUTP nick end labeling (TUNEL). Harvested myocardium was embedded in optimal cutting temperature compound (Tissue‐Tek, Torrance, CA, USA) and cut into 10‐μm‐thick sections with a cryostat. Isolated myocytes were fixed with 4% paraformaldehyde for 1 h. Then, the apoptosis of cardiac tissue slides and isolated myocytes were detected with TUNEL assay kits (Abcam, Cambridge, MA, USA; Roche) as per the manufacturer's instructions. Cell nuclei were tagged by 4′,6‐diamidino‐2‐phenylindole (DAPI; Abcam). Myocytes on cardiac tissue slides were tagged by cardiac troponin I (conjugated with Alexa Fluor 488; Abcam). The fluorescence images were captured by an inverted fluorescence microscope and further analyzed by software imagej (version 1.38; NIH, Bethesda, MD, USA).

### *In situ* reactive oxygen species detection

*In situ* reactive oxygen species (ROS) detection *in vivo* and *in vitro* was carried out according to our previous studies with dihydroethidium (DHE) staining 16. Briefly, frozen tissue sections and isolated myocytes were incubated with DHE (10 μmol·L^−1^; Beyotime, Shanghai, China) at 37 °C for 45 min in a humidified dark chamber. Then the samples were observed with an inverted fluorescence microscope. The images were captured and analyzed with software imagej (NIH).

### Western blotting

The whole‐cell extracts from harvested myocardium and cultured myocytes were prepared with a cell lysis buffer system (Santa Cruz Biotechnology, Dallas, TX, USA). Total protein and nuclear protein were extracted with Total Protein Extraction Reagents (Beyotime) and Nuclear Extraction Reagents (Beyotime), respectively, in accordance with the protocol provided by the manufacturer. A BCA kit (Pierce, Carlsbad, CA, USA) was used to measure the protein concentrations. Thirty micrograms of protein was loaded and separated by vertical SDS/PAGE. Then the protein was transferred electrically to poly(vinylidene difluoride) membranes. The membranes were incubated with primary antibodies against MKK3 (Sigma‐Aldrich; 1 : 500), phospho‐MKK3 (Sigma‐Aldrich; 1 : 500), MKK6 (Sigma‐Aldrich; 1 : 500), phospho‐MKK6 (Sigma‐Aldrich; 1 : 500), p38 (Cell Signaling Technology; 1 : 250), phospho‐p38 (Cell Signaling Technology, Danvers, MA, USA; 1 : 250), active caspase‐3 (Abcam; 1 : 1000), p65 (Abcam; 1 : 1000), histone H3 (Abcam; 1 : 1000) and glyceraldehyde 3‐phosphate dehydrogenase (GAPDH; Abcam; 1 : 500) at 4 °C for 10 h. Then the membranes were washed by Tris‐buffered saline–Tween‐20 (0.02%, TBST). Horseradish peroxidase‐conjugated secondary antibodies were used to incubate the membranes at room temperature for 2 h. SuperSignal West Pico chemiluminescence reagent (Thermo Fisher Scientific, Carlsbad, CA, USA) was used to develop the membranes, and the immunoblots were visualized on X‐ray films. The software imagej was used to quantify and analyze the densities of the blots.

### Immunoprecipitation

In this study, the association between ASK1 and Trx was assessed by immunoprecipitation (IP), which was carried out as previously described 17. Briefly, 400 μg whole‐cell extract was pre‐cleared by incubating with 5 μg normal rabbit serum plus protein A/G‐agarose beads at 4 °C on a rotator for 10 h. Then 5 μg first protein (Trx)‐specific antiserum was used to incubate the extracts with 50 μL protein A/G‐agarose beads for 2 h. After centrifugation at 14 000 ***g*** for 10 min and washing with lysis buffer, the immune complexes were harvested after each IP. SDS/PAGE was carried out to separate the immune complexes, which were then detected by immunoblotting (Immobilon‐P; Millipore, Burlington, MA, USA) with the second protein (ASK1)‐specific antibody. The band intensities were quantified and analyzed by the software imagej (version 1.38; NIH).

### ASK1 kinase activity determination

The ASK1 kinase activity was determined with the method described previously with some modifications 18, 19. Briefly, the immunocomplex‐bonded A/G‐agarose beads mentioned above were washed with kinase buffer (20 mmol·L^−1^ Tris/HCl; 20 mmol·L^−1^ MgCl_2_; pH 7.4) three times. Then these beads were incubated with glutathione *S*‐transferase (GST)–MKK6 fusion protein (Millipore) at 30 °C for 15 min in a final volume of 25 μL kinase buffer containing ATP at 100 μmol·L^−1^. Harvested samples were subjected to SDS/PAGE. The expression of phosphorylated MKK6 was detected with primary antibody phospho‐MKK6 (Sigma‐Aldrich; 1 : 500) by following the protocol described in the western blotting section.

### Nicotinamide adenine dinucleotide 2′‐phosphate (NADPH) assay

The reduced tetrasodium salt hydrate NADPH levels were determined in homogenates of cardiac tissue and isolated myocytes by using an NADP/NADPH assay kit (Sigma‐Aldrich) as per the manufacturer's instructions.

### Statistical analysis

Data collected in this study are presented in the mean ± SD. Student's *t* test and one‐way ANOVA were used to analyze the differences between groups. The *post hoc* test was performed by Bonferroni adjustments. The software spss statistics (version 22.0; IBM Corp., Armonk, NY, USA) was used to analyze the data. The differences were considered statistically significant at *P* < 0.05.

## Results

### AGI‐1067 improved AGE‐induced cardiac dysfunction

**Figure 1:**
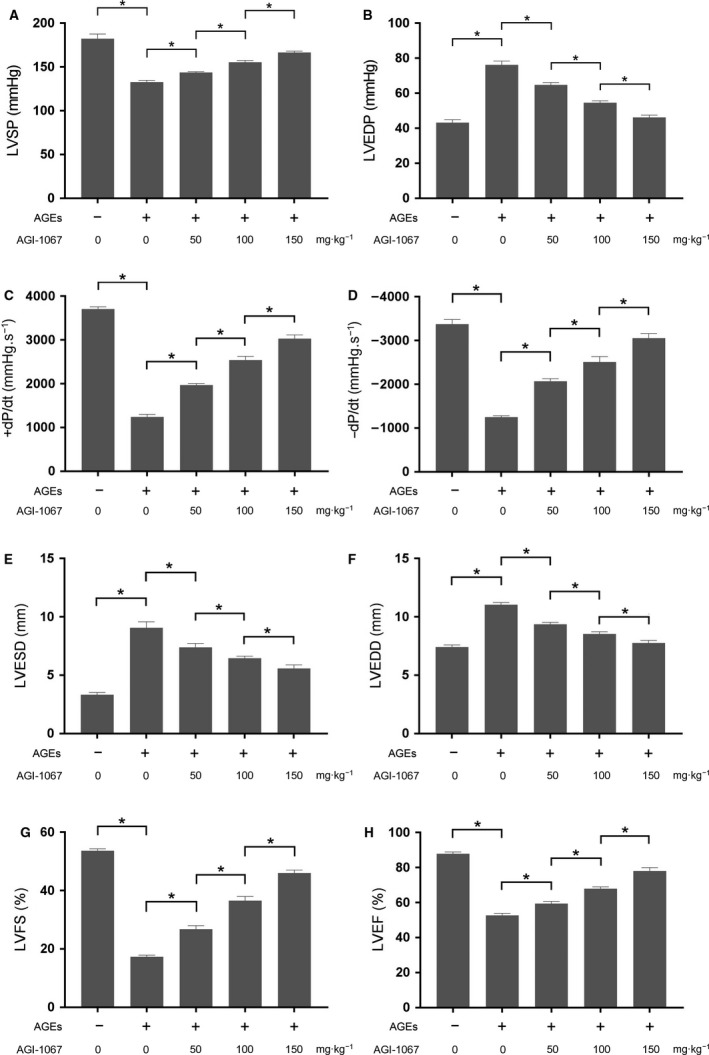
The measured LVSP, LVEDP, maximal rate of increased pressure (+LVd*P*/d*t*), maximal rate of decreased pressure (−LVd*P*/d*t*), LVESD, LVEDD, LVFS and LVEF in animals that received AGE exposure and/or AGI‐1067 administration at dosages of 50, 100 and 150 mg·kg^−1^ are shown in (A)–(H), respectively. The results are expressed as the mean ± SD of six independent experiments. Differences between groups were analyzed by one‐way ANOVA. *Differences were statistically significant (*P* < 0.05).

The animals were sacrificed 4 weeks after being exposed to AGEs. As shown in Fig. 1, the results from the invasive hemodynamic and echocardiographic measurements showed that the LVEDP, LVEDD and LVESD were increased while LVSP, +LVd*P*/d*t*, −LVd*P*/d*t*, LVEF and LVFS were decreased significantly in AGE‐exposed animals compared with control animals. These results indicated that in this study, the exposure to AGEs impaired both systolic and diastolic cardiac functions. AGI‐1067 co‐administration significantly improved the diastolic dysfunction by improving both hemodynamic and echocardiographic parameters in AGE‐exposed animals in a concentration‐dependent manner.

### AGI‐1067 attenuated apoptosis of AGE‐exposed myocardium and cultured myocytes

**Figure 2:**
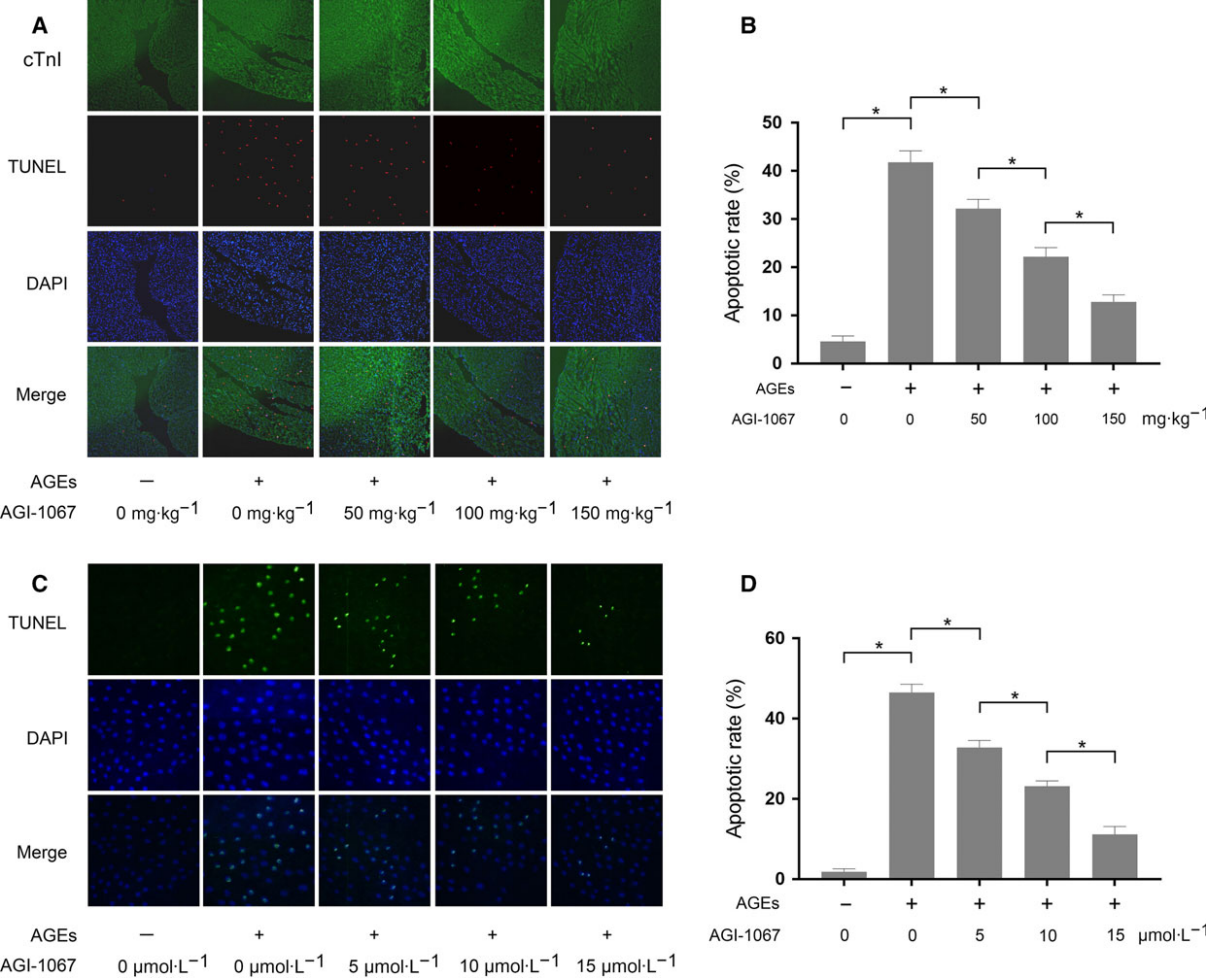
(A) Captured fluorescence images of cardiac troponin I (cTnI), TUNEL, DAPI and their merged images of cardiac tissue sections. (B) The apoptotic rate as a percentage in cardiac tissue from animals that received AGE exposure and/or AGI‐1067 administration at dosages of 50, 100 and 150 mg·kg^−1^. (C) Captured fluorescence images of TUNEL, DAPI and their merged images of cultured primary myocytes. (D) The apoptotic rate as a percentage in primary myocytes that received AGE exposure and/or AGI‐1067 treatment at concentrations of 5, 10 and 15 μmol·L^−1^. The results are expressed as the mean ± SD of six independent experiments. Differences between groups were analyzed by one‐way ANOVA. *Differences were statistically significant (*P* < 0.05).

TUNEL assay‐detected apoptosis in both myocardium and cultured myocytes is shown in Fig. 2. Compared with control, the apoptosis increased dramatically in both AGE‐exposed myocardium and cultured myocytes, indicating AGEs induced contractile unit lost. However, the co‐administration of AGI‐1067 significantly inhibited the AGE‐induced cardiac apoptosis *in vivo* and *in vitro* in a concentration‐dependent manner.

### AGI‐1076 inhibited ROS generation in AGE‐exposed myocardium and cultured myocytes

**Figure 3:**
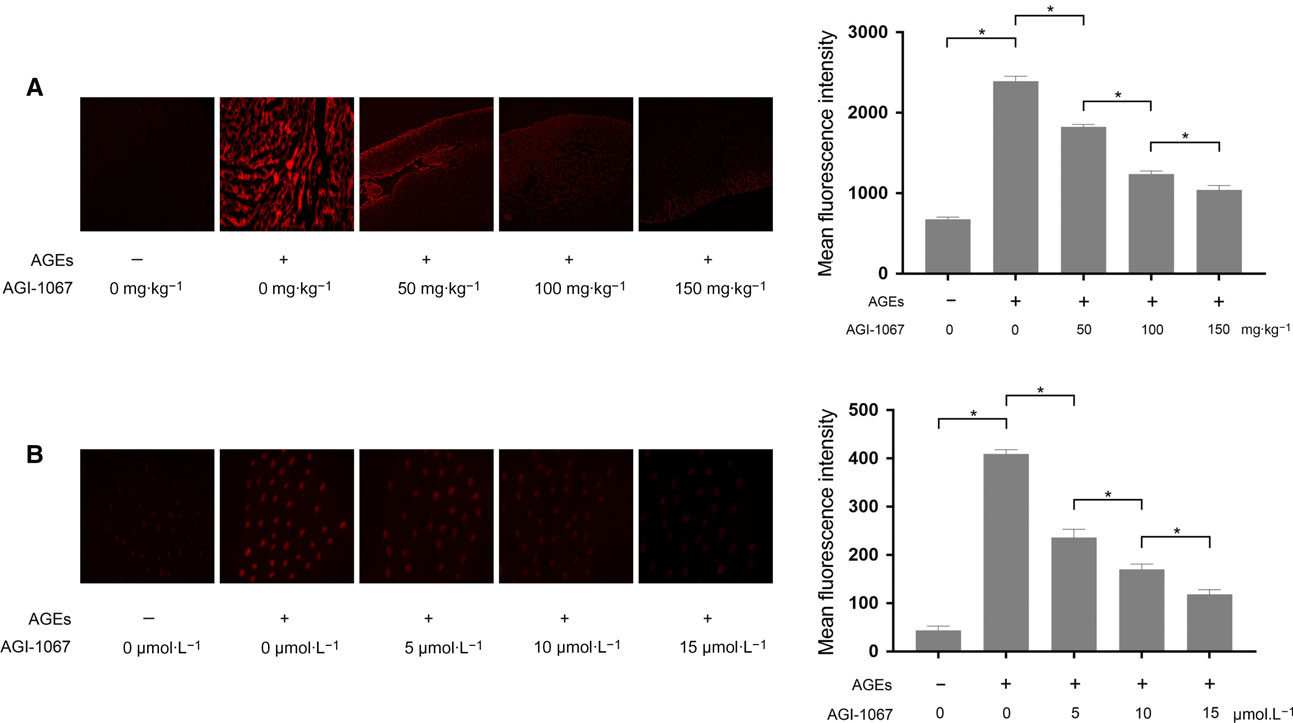
(A) Left, the captured fluorescence images of DHE‐stained cardiac cryostat sections from animals exposed to AGEs and/or AGI‐1067 administration at dosages of 50, 100 and 150 mg·kg^−1^. Right, the mean fluorescence intensity (MFI) of the DHE staining. (B) Left, the captured fluorescence images of DHE‐stained isolated myocytes that received AGE exposure and/or AGI‐1067 treatment at concentrations of 5, 10 and 15 μmol·L^−1^. Right, the MFI of the DHE staining. The results are expressed as the mean ± SD of six independent experiments. Differences between groups were analyzed by one‐way ANOVA. *Differences were statistically significant (*P* < 0.05).

ROS generation was detected by the probe DHE. As shown in Fig. 3, the ROS generation levels were found to be dramatically increased in AGE‐exposed myocardium and cultured myocytes. AGI‐1067 administration, however, reduced ROS formations in AGE‐exposed myocardium and cultured myocytes in a concentration‐dependent manner.

### AGI‐1067 reduced activation of nuclear factor κB and NADPH activation in AGE‐exposed myocardium and cultured myocytes

**Figure 4:**
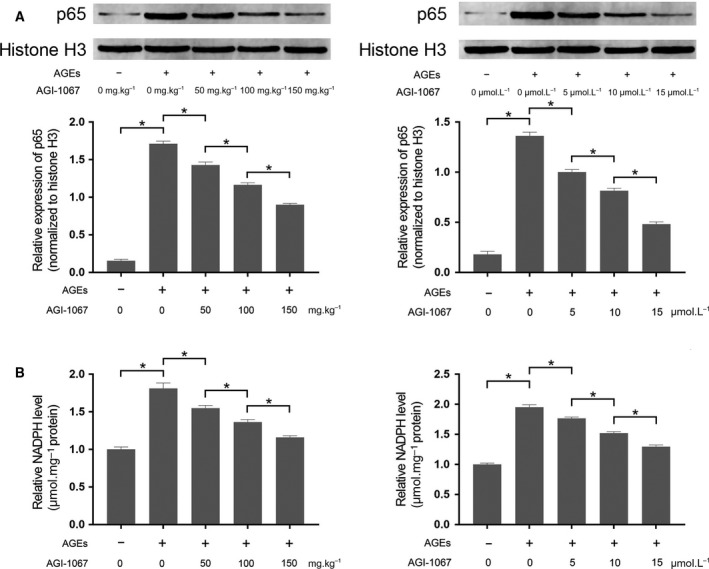
(A) Top, immunoblots of p65 and histone H3 in cardiac tissue and isolated myocytes, respectively. Bottom, the relative expression level of p65 in nuclear protein in cardiac tissue and isolated myocytes exposed to AGEs and/or AGI‐1067 administration. (B) The relative NADPH levels in cardiac tissue and isolated myocytes exposed to AGEs and/or AGI‐1067 administration. The results are expressed as the mean ± SD of six independent experiments. Differences between groups were analyzed by one‐way ANOVA. *Differences were statistically significant (*P* < 0.05).

Nuclear factor κB (NF‐κB) activation was assessed by the nuclear translocation of p65, measured with p65 western blotting in nuclear extracts with the nuclear protein histone H3 introduced as the internal reference. As shown in Fig. 4, the AGE treatment increased p65 nuclear translocation as well as the NADPH levels in both AGE‐exposed myocardium and cultured myocytes. However, the administration of AGI‐1067 inhibited the nuclear translocation of p65 and reduced NADPH levels in the AGE‐exposed myocardium and cultured myocytes in a concentration‐dependent manner.

### AGI‐1067 suppressed the activation of MKKs/p38 MAPK signaling pathway in AGE‐exposed myocardium and cultured myocytes

**Figure 5:**
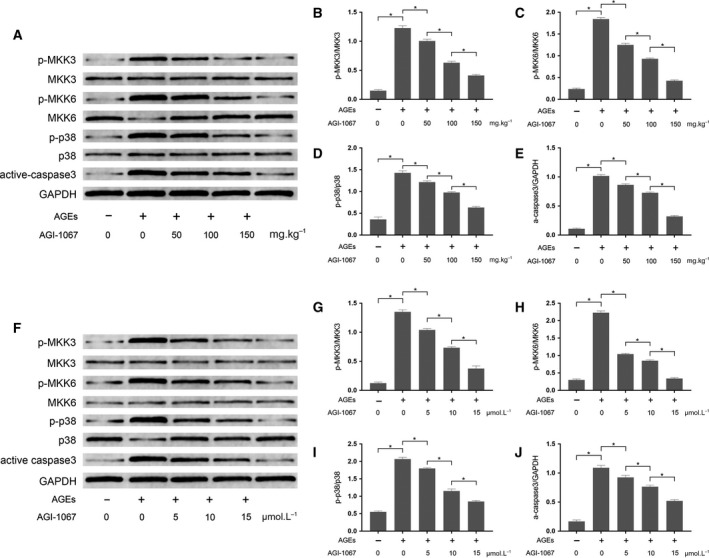
(A) The immunoblots of p‐MKK3, MKK3, p‐MKK6, MKK6, p‐p38, p38, active caspase‐3 and GAPDH in cardiac tissue. (B–E) Phosphorylation level of MKK3 (B), MKK6 (C) and p38 (D), and expression level of active caspase‐3 (E) in cardiac tissue from animals that received AGE exposure and/or AGI‐1067 administration at dosages of 50, 100 and 150 mg·kg^−1^. (F) Immunoblots of p‐MKK3, MKK3, p‐MKK6, MKK6, p‐p38, p38, active caspase‐3 and GAPDH in cultured primary myocytes. (G–J) Phosphorylation level of MKK3 (G), MKK6 (H) and p38 (I), and expression level of active caspase‐3 (J) in primary myocytes that received AGE exposure and/or AGI‐1067 treatment at concentrations of 5, 10 and 15 μmol·L^−1^. The results are expressed as the mean ± SD of six independent experiments. Differences between groups were analyzed by one‐way ANOVA. *Differences were statistically significant (*P* < 0.05).

The activation of MKKs/p38 MAPK apoptotic signaling was evaluated by analyzing the immunoblots. As shown in Fig. 5, the phosphorylation of MKK3, MKK6 and p38 MAPK were up‐regulated in AGE‐exposed myocardium and cultured myocytes. As a result, the expression of the pro‐apoptotic protein caspase‐3 (active form) increased dramatically. However, AGI‐1067 treatment down‐regulated the phosphorylation of MKK3, MKK6 and p38 MAPK, as well as the expression levels of caspase‐3 *in vivo* and *in vitro* in a concentration‐dependent manner.

### By strengthening the ASK1‐Trx1 association, AGI‐1067 impaired ASK1 activation in AGE‐exposed myocardium and cultured myocytes

**Figure 6:**
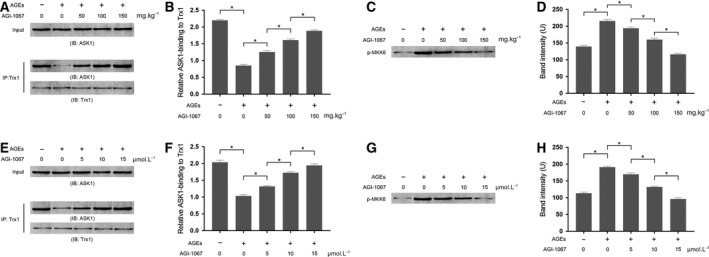
(A) The results of IP that evaluated the association between ASK1 and Trx1. Cardiac tissue extract was immunoprecipitated with antibodies against Trx1. (B) Relative Trx1‐bound ASK1 was quantified by comparing pulldown ASK1/input ASK1 in cardiac tissue exposed to AGEs and/or AGI‐1067 treatment. (C) Immunoblots of p‐MKK6 that resulted from GST–MKK6 incubated with the Trx1–ASK1 immunocomplex extracted from cardiac tissue. (D) The band intensity of p‐MKK6. (E) The results of IP assessing the association between ASK1 and Trx1 in cultured primary myocytes. (F) The relative ASK1‐binding to Trx1 in myocytes exposed to AGEs and/or AGI‐1067 incubation. (G) Immunoblots of p‐MKK6 that resulted from GST–MKK6 incubated with the Trx1–ASK1 immunocomplex extracted from cultured primary myocytes. (H) The band intensity of p‐MKK6. The results are expressed as the mean ± SD of six independent experiments. Differences between groups were analyzed by one‐way ANOVA. *Differences were statistically significant (*P* < 0.05).

The results of IP and ASK1 enzymatic activity are shown in Fig. 6. In both myocardium and primary myocytes, the AGE treatment dramatically facilitated the separation of the Trx1–ASK1 complex, which would further promote the activation of ASK1. AGI‐1067 administration, however, inhibited the disassociation of Trx1 from ASK1 in a concentration‐dependent manner. By assessing the Trx1–ASK1 complex‐induced phosphorylation of GST–MKK6, the enzymatic activity of ASK1 was determined. AGEs significantly increased the Trx1–ASK1 complex‐induced phosphorylation of GST–MKK6 *in vivo* and *in vitro*, which was impaired by AGI‐1067 treatment in a concentration‐dependent manner.

## Discussion

With the rapidly increasing mortality and morbidity of diabetes in the past few decades worldwide, diabetic complications have attracted our attention. Uncontrolled and sustained hyperglycemia‐associated pathological changes are the pivotal characters of diabetic metabolic disorders. The occurrence, development and progression of cardiac dysfunction are featured clinical manifestations of diabetic cardiovascular complications 20. In responses to diabetic metabolic disorders, responses of the heart include aberrant gene expression, subcellular defects and apoptosis 20. Our and others' previous investigations proved that cardiomyocyte apoptosis is a hallmark of DbCM and responsible for the development of impaired cardiac functions 9, 21, 22. Fostered during diabetes, AGEs induce and facilitate many diabetic pathological processes. It has been established in several recent studies that increased level of AGEs was highly correlated with reduced heart functions and could be considered as a biomarker predicting the prognosis of congestive heart failure 6, 23, 24. In the current study, AGEs were used to treat rats via intraperitoneal injections. Hemodynamic results showed that both the systolic and diastolic cardiac functions were significantly impaired. More apoptotic myocytes were observed in myocardium exposed to AGEs. Moreover, increased apoptotic events were identified in AGE‐incubated primary myocytes, which was consistent with previous reports.

Although the mechanisms of apoptosis are complex, several previous investigations pointed out that activation of signaling by several nuclear factors, such as p38 MAPK and NF‐κB, is involved 25. The static status is maintained when p38 MAPK binds to its inhibitor, Keap1. When challenged by harmful stimuli, p38 MAPK and NF‐κB would be activated and further trigger their targeted gene transcription or modification 26. It was reported that activation of p38 MAPK and NF‐κB induced caspase‐3 activation, leading to cell death 8, 27. It has been well established that the activation of p38 MAPK is regulated by its upstream kinases, the MKKs 28. Specifically, among the MKKs, MKK3 and MKK6 were reported to regulate activation of p38 MAPK 29. In the current study, we found that in both myocardium and primary myocytes exposed to AGEs, the phosphorylation level of MKK3 and MKK6 increased significantly. As a result, the p38 MAPK was activated by increased phosphorylation, leading to the activation of the apoptotic effector, caspase‐3. Thus, activation of MKKs would be an ideal molecular target for agents attenuating myocyte apoptosis.

ASK1 is believed to be a critical activator of MKKs, and is also referred as a MAPKKK 29. By binding to the N terminus of ASK1, the Trx1–ASK1 complex is formed and the activation of ASK1 is inhibited 30. Previous studies described that AGEs could trigger activation of ASK1 by facilitating the disassociation of the Trx1–ASK1 complex 31. Moreover, AGEs were also identified as the activators of NF‐κB 32. In this study, we also found that AGE exposure facilitated the nuclear translocation of NF‐κB as well as disassociation of the Trx1–ASK1 complex, which resulted in ASK1 activation *in vivo* and *in vitro*. AGI‐1067 is the monosuccinic acid ester of the antioxidant probucol 33. Previous studies revealed the potent antioxidant effect of AGI‐1067 34. Results from the current study showed that AGI‐1067 reduced AGE‐mediated ROS generation both *in vivo* and *in vitro*. The activation of NADPH oxidase was suppressed by AGI‐1067 administration. Moreover, we also found that by blocking the disassociation of Trx1 and ASK1, AGI‐1067 inhibited the AGE‐induced ASK1 activation *in vivo* and *in vitro*. Correspondingly, as the downstream signaling pathway of ASK1, the MKKs/p38 MAPK/caspase pathway was shut down by AGI‐1067. As a result, AGI‐1067 administration decreased apoptosis in both myocardium and cultured myocytes exposed to AGEs. The cardiac functions were improved by AGI‐1067 due to reduced AGE‐induced myocardial apoptosis.

In this study, we provided evidence indicating that excessive ROS were generated and the ASK1/MKKs/p38 MAPK and NF‐κB apoptotic signaling pathways were activated in AGE‐exposed myocardium and isolated myocytes. The administration of ASK1 inhibitor AGI‐1067 dramatically improved AGE‐induced diastolic and systolic cardiac dysfunctions. Inhibiting of activation of this apoptotic pathway was involved in the cardioprotective effects of AGI‐1067. Several previous clinical trials such as the Aggressive Reduction of Inflammation Stops Events (ARISE) study showed that AGI‐1067 failed to meet the primary endpoint of relative risk reduction 35. These studies concentrated on the anti‐atherosclerotic activity. However, until now, there are still no trials designed to investigate the therapeutic effect of AGI‐1067 on heart failure of DbCM. Our study provided such clues, indicating this possible pharmacological value of AGI‐1067. Of course, these are just theories proposed from our preliminary data, and more rigorous and dedicated clinical trials are needed to test them and predict the value of clinical application of AGI‐1067 in the future.

## Author contributions

ZL, SZ and YG contributed to the study design and concept. ZL, SZ and XW implemented the experiments. CQ analyzed the data. ZL wrote the manuscript. YG revised the manuscript. All authors have read and approved the final version of the manuscript.
